# Decreased Tongue–Lip Motor Function in Japanese Population with Low Taste Sensitivity: A Cross-Sectional Study

**DOI:** 10.3390/jcm13164711

**Published:** 2024-08-11

**Authors:** Tomomi Yata, Norio Aoyama, Toshiya Fujii, Sayuri Kida, Kentaro Taniguchi, Taizo Iwane, Katsushi Tamaki, Masato Minabe, Motohiro Komaki

**Affiliations:** 1 Department of Periodontology, Kanagawa Dental University, 82 Inaoka-cho, Yokosuka 238-8580, Kanagawa, Japan; t.oct.o2m6o@gmail.com (T.Y.); t.fujii@kdu.ac.jp (T.F.); kdsyr61@gmail.com (S.K.); tanikentaniken2@gmail.com (K.T.); m.komaki@kdu.ac.jp (M.K.); 2 Department of Education Planning, Kanagawa Dental University, 82 Inaoka-cho, Yokosuka 238-8580, Kanagawa, Japan; 3 Center for Innovation Policy, Graduate School of Health Innovation, Kanagawa University of Human Services, 3-25-10 Tonomachi, Kawasaki-ku, Kawasaki 210-0821, Kanagawa, Japan; t.iwane-c2p@kuhs.ac.jp; 4 Department of Functional Recovery of TMJ and Occlusion, Kanagawa Dental University, 82 Inaoka-cho, Yokosuka 238-8580, Kanagawa, Japan; tamaki@kdu.ac.jp; 5 Bunkyou Dori Dental Clinic, 2-4-1 Anagawa, Inage-ku, Chiba 263-0024, Chiba, Japan; minabe-m@wk9.so-net.ne.jp; 6 Department of Environmental Pathology, Kanagawa Dental University, 82 Inaoka-cho, Yokosuka 238-8580, Kanagawa, Japan

**Keywords:** super aging society, taste disorder, oral function, oral frailty, quality of life

## Abstract

**Background/Objectives**: Taste disorders have a negative impact on meal enjoyment, which is essential for maintaining adequate nutrition and quality of life. Japan is a rapidly aging society with an increasing number of individuals with taste disorders. However, despite the increasing prevalence of taste disorders, the correlation between oral frailty and taste sensitivity remains largely unknown. The objective of this study was to assess the relationship between oral health status and taste sensitivity among the Japanese population. **Methods**: Participants were recruited from Kanagawa Dental University Hospital Medical–Dental Collaboration Center between 2018 and 2021. The exclusion criteria were severe systemic infections, pregnancy, or lactation. Clinical examinations, oral function assessments, and taste tests were conducted using tap water and 1% sweet, 0.3% salty, 0.03% umami, and 0.1% umami tastants. The relationships between oral function, systemic indicators, and taste sensitivity were statistically evaluated. **Results**: Of the 169 participants included in this cross-sectional study, 39.6% were male and 60.4% were female (median age, 68 years). Participants with low taste sensitivity showed a decline in tongue–lip motor function, independent of age, sex, or smoking status. A multiple logistic regression analysis conducted using two age categories—younger than 65 years and older than 65 years—revealed an association between tongue–lip motor function and taste sensitivity among participants younger than 65 years. **Conclusions**: Decreased taste sensitivity is associated with tongue–lip motor function. Therefore, the early maintenance of oral function and taste sensitivity may be beneficial for optimal tongue–lip motor function.

## 1. Introduction

In Japan, the percentage of the total population aged 65 years or older has increased from 4.9% in 1950 to 28.6% in 2020 and is expected to further increase to 34.8% by 2040 [1]. Against a background of an increasing incidence of lifestyle-related diseases and an aging society, the number of patients who visit otorhinolaryngologists with complaints of taste disorders is increasing every year [2]. A 2019 survey conducted in Japan indicated that 270,000 patients with taste disorders seek medical attention annually; however, the treatment of taste disorders is not covered by health insurance in Japan [3]. The symptoms of taste disorders include quantitative taste disorders, such as hypogeusia and ageusia, and qualitative taste disorders, such as phantogeusia, parageusia, and cacogeusia [4]. The loss of meal enjoyment caused by a decreased sense of taste, resulting in a loss of appetite, may affect an individual’s health status [5,6].

Previous studies have demonstrated that oral function is associated with physical frailty, sarcopenia, subsequent disability, and a shortened healthy lifespan [7,8]. Maintaining the health and independent living of elders improves their quality of life and benefits the society as a whole, particularly in light of the rapidly increasing medical and nursing care costs worldwide [9,10].

In 2016, “oral hypofunction” was proposed as the stage at which administration of dental treatment can result in recovery before oral dysfunction occurs. The Japanese Society of Gerodontology indicated the diagnostic criteria for oral hypofunction [11]. In 2023, Tanaka et al. proposed a simplified assessment method for oral frailty [12]. The early detection of the frailty cycle, which begins with a decline in oral function and taste, and the timely initiation of dental interventions are expected to contribute to an increase in healthy life expectancy. Oral function directly affects dietary intake, and the intake of tasty food is essential for healthy living. However, scientific data on the association between decline in taste sensitivity and oral health are limited. Therefore, we conducted this observational study to determine the association between poor oral health and low taste sensitivity among the Japanese population.

## 2. Materials and Methods

### 2.1. Study Population

The participants were recruited for inclusion in this cross-sectional study from the Medical and Dental Collaboration Center in Kanagawa Dental University Hospital between 2018 and 2021. The inclusion criteria were as follows: age ≥ 20 years and consent to participate in this study. The exclusion criteria were severe systemic infection, pregnancy, and lactation. The ethics committee of the School of Dentistry at Kanagawa Dental University approved this study (approval no.: 801). The study protocol conformed to the principles of the 1975 Declaration of Helsinki, which were revised in 2013. Each participant was informed of the study aim and formalities, and written informed consent was obtained from all the participants prior to their participation in the study.

### 2.2. Clinical Examinations

All examinations were performed at the Center for Medical and Dental Collaboration in Kanagawa Dental University Hospital. Body composition analysis was performed using body indices such as height, weight, and body mass index (BMI). Body composition analysis was performed using an analyzer (InBody 460; InBody Japan, Tokyo, Japan), and body indices such as height, weight, and body mass index (BMI) were recorded. The participants were classified into two categories based on the criteria outlined by the Japan Society for Human Health Study: BMI ≥ 18.5 kg/m^2^ and BMI < 18.5 kg/m^2^ [13]. Two trained periodontists (N. A. and M. M.) counted the number of teeth present (excluding the third molars) for each participant [14,15,16]. General baseline data, such as age and sex, were extracted from the medical records of the participants.

### 2.3. Taste Sensitivity Test

Five tastants were prepared: tap water, 1% sweet (Tea Time Mate^®^: Mitsui DM Sugar Co., Ltd., Tokyo, Japan), 0.3% salty (Hakata no Shio^®^ Baked Salt: HAKATA SALT Co., Ltd., Ehime, Japan), 0.03% umami (AJI-NO-MOTO^®^: Ajinomoto Co., Inc., Tokyo, Japan), and 0.1% umami (AJI-NO-MOTO^®^: Ajinomoto Co., Inc., Tokyo, Japan). The whole-mouth taste test method was used in this study.

The participants were instructed to rinse their mouths before and after each test. One milliliter of each test solution was put in mouth using a disposable paper cup. Participants were required to report whether they “felt the taste” or “not”. If they chose “felt the taste”, they were then required select what they tasted from the following six choices: “sweet”, “salty”, “sour”, “bitter”, “Dashi (soup stock containing umami)” or “undefined” tastes. We determined whether each participant’s taste selection was correct or not.

### 2.4. Oral Function Tests

Oral functional decline was defined based on the values proposed by the Japanese Society of Gerodontology [11,17].

#### 2.4.1. Oral Hygiene

The number of microorganisms on the dorsum of the tongue was measured by rubbing a cotton swab on the central area of the dorsum and placing the collected specimen in a bacterial counter (PHC Co., Ltd., Tokyo, Japan). A total number of microorganisms of ≥3.162 × 106 CFU/mL was considered indicative of poor oral hygiene.

#### 2.4.2. Oral Dryness

Mucosal wetness was measured using an oral moisture-checking device (Mucus, Life Co., Ltd., Saitama, Japan), which was applied to the central area of the dorsum of the tongue. The measurement was repeated three times, and the median of the three measurements was used for analysis. An oral dryness score below 27.0 was classified as oral dryness.

#### 2.4.3. Occlusal Force

Occlusal force was measured by asking the participants to clench their teeth for 3 s in habitual occlusion with a thin indicator sheet covering the entire dentition (Dental Pre-scale II, GC Corporation, Tokyo, Japan). Occlusal force was measured electronically and a value lower than 200 N was classified as low occlusal force.

#### 2.4.4. Tongue–Lip Motor Function

Tongue–lip motor function was evaluated based on motor speed, which was quantified using the oral diadochokinesis (ODK) score. The ODK score represents the number of times the participant is able to repeat each of the /pa/, /ta/, and /ka/ syllables in 5 s. ODK was measured using an automatic counter (Kenkokun Handy; Takei Scientific Instruments Co., Ltd., Niigata, Japan). Repetition of the /pa/, /ta/, or /ka/ syllables less than six times per second was classified as poor tongue–lip motor function.

#### 2.4.5. Tongue Pressure

Maximum tongue pressure was assessed using a JMS tongue pressure measurement device (TPM-01, JMS Co., Ltd., Tokyo, Japan). Each participant was asked to compress the balloon of the device between the tongue and anterior palate using the maximum voluntary force of the tongue. Tongue pressure was considered impaired if the maximum tongue pressure was less than 30 kPa.

#### 2.4.6. Masticatory Function

Masticatory function was assessed based on the glucose concentration obtained from chewing a gummy jelly. The participants were asked to chew a test glucose-containing gummy jelly for 20 s without swallowing it, hold 10 mL of water in their mouth, and spit the water out together with the jelly. The amount of glucose eluted from the chewed jelly into the water was measured in mg/dL using a Gluco Sensor GS-II (GC Corporation, Tokyo, Japan). A glucose level of <100 mg/dL was considered indicative of decreased masticatory function.

#### 2.4.7. Swallowing Function

A self-administered questionnaire, the 10-item Eating Assessment Tool (EAT-10, Nestlé, Vevey, Switzerland), was used to assess swallowing function. An EAT-10 score of three or higher was considered indicative of poor swallowing function.

### 2.5. Statistical Analysis

The Kolmogorov–Smirnov test was used to test the normality of the data distributions. Numerical data with skewed distributions were presented as medians and interquartile ranges. Systemic status (age, height, and body weight) and number of teeth were compared using the Mann–Whitney U test. The sex, BMI, current smoking status, and oral status distributions in the correct and incorrect taste groups were analyzed using Fisher’s exact test. In addition, multiple logistic regression analysis was performed with taste sensitivity as the objective variable; tongue and lip motor function as the explanatory variables; and age, sex, BMI, current number of teeth, and current smoking status as adjustment variables. A multiple logistic regression analysis was performed with all adjustment variables entered except age. Additionally, participants were stratified according to age (<65 and ≥65 years old) for subgroup analysis. All statistical analyses were performed using JMP version 17.0 (Subscription) Installer (SAS Institute Inc., Cary, NC, USA). The statistical significance was set at *p* < 0.05.

## 3. Results

**Figure 1:**
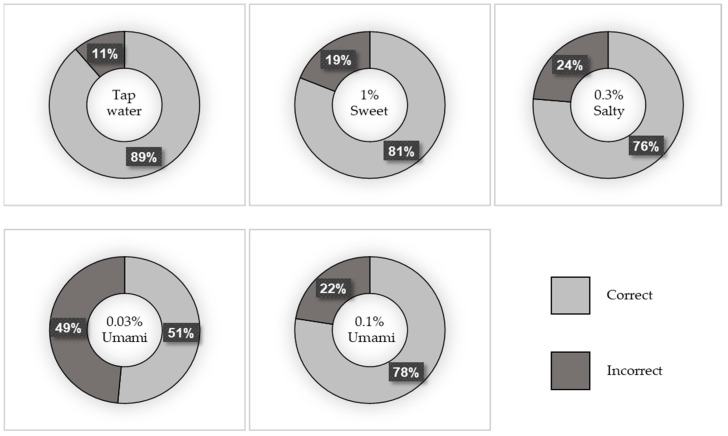
Percentage of correct taste tests.

**Figure 2:**
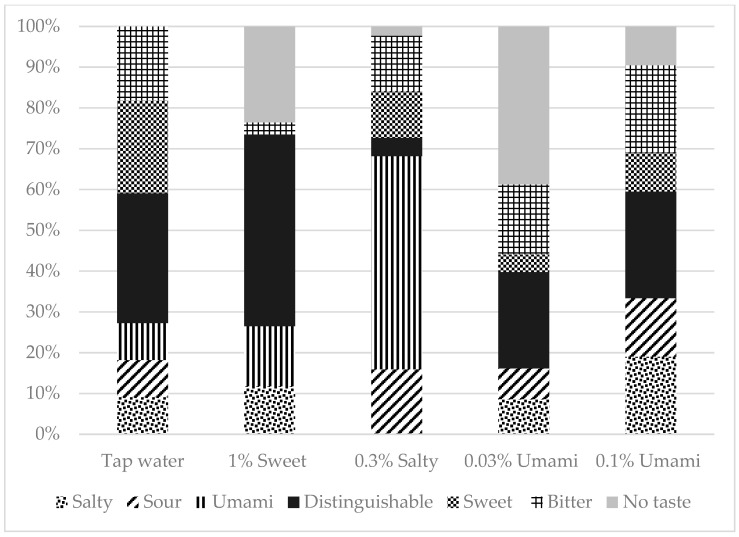
Taste tests Breakdown of false answers.

A total of 169 participants were included in this study. Of these, 39.6% were men and 60.4% were women. The median age (IQR) of the participants was 68 (58–76) years. Figure 1 depicts the percentage of correct taste tests, and Figure 2 shows the breakdown of incorrect answers. The characteristics of the study population are summarized in Table S1. There were no significant differences in BMI, number of teeth, or current smoking status between the correct and incorrect groups for any of the test liquids. However, the participants in the incorrect salt test group were significantly older than those in the correct salt test group (*p* = 0.001).

Table S2 shows the association between taste sensitivity and oral function status. The incorrect 1% sweet (*p* = 0.017) and 0.3% salty (*p* = 0.012) test groups had significantly more participants with poor tongue–lip motor function than the correct groups. No significant association between taste sensitivity and oral dryness was observed in this study.

In addition to Table S1, Table 1 shows the results of the multiple logistic regression analysis of incorrect taste sensitivity results adjusted for age, sex, BMI, number of teeth, and current smoking status. This model showed that reduced sensitivity to sweet taste was independently associated with poor tongue–lip motor function (odds ratio [OR], 3.15; 95% confidence interval [CI], 1.12–8.84) and hypo ODK /pa/ (OR, 4.32; 95% CI, 1.70–10.99). Sensitivity to 0.1% umami taste was independently associated with hypo ODK/ta/ (OR, 2.98; 95% CI, 1.22–7.30).

Table 2 shows the results of the multiple logistic regression analysis of the relationship between poor tongue–lip motor function and low taste sensitivity using two age categories: <65 years and ≥65 years old. The results showed that tongue–lip motor function was not associated with taste sensitivity in ≥65 years old participants. However, a decreased sensitivity to sweet taste was independently associated with poor tongue–lip motor function (OR, 7.47; 95% CI, 1.35–41.48) and hypo ODK /pa/ (OR, 30.85; 95% CI, 3.76–252.83). Sensitivity to salty taste was independently associated with poor tongue–lip motor function (OR, 6.14; 95% CI, 1.04–36.17) and hypo ODK /ta/ (OR, 14.28; 95% CI, 1.77–115.05). Furthermore, sensitivity to 0.1% umami taste was independently associated with poor tongue–lip motor function (OR, 5.46; 95% CI, 1.01–29.36), hypo ODK /ta/ (OR, 11.98; 95% CI, 1.32–108.65), and hypo ODK /ka/ (OR, 10.50; 95% CI, 1.60–69.06).

## 4. Discussion

In this study, we evaluated physical and oral statuses to identify whether impaired oral status is associated with taste sensitivity. The results showed that tongue–lip motor function was not associated with taste sensitivity in ≥65 years old participants. However, tongue–lip motor function was associated with taste sensitivity among participants younger than 65 years. Moreover, participants with low taste sensitivity showed a decline in tongue–lip motor function, independent of age, sex, or smoking status.

Taste recognition is determined using both chemical and electrical stimuli [18]. Taste testing using solutions, a chemical stimulation method, is a reliable method of assessing taste recognition [19]. Solution-based taste tests include whole-mouth and topical methods [20]; however, no standardized technique has been established for whole-mouth taste tests. A previous study showed that whole-mouth taste tests can determine general taste sensitivity [21]. Thus, we adopted this method and included five taste qualities—tap water and 1% sweet, 0.3% salty, 0.03% umami, and 0.1% umami tastes—based on the information in previous studies [22,23,24,25]. However, it should be noted that previous studies have indicated that taste recognition threshold values vary according to the volume of the testing liquid used [26] and environment where the test was conducted [27].

We found that the ages the participants in the correct and incorrect 0.3% salty test groups differed significantly (Table S1). Previous studies have shown an association between aging and a decrease in taste sensitivity [28,29]. A systematic review regarding the effects of aging on taste function indicated that taste perception declines with age [30]. However, the reported extent of this decline differs across studies [31]. Taste sensation may decline with age because there are fewer taste papillae and buds and alterations in afferent nerve signaling to the brain [32].

The results of this study showed that the male participants in the 1% sweet test groups had significantly lower taste sensitivity than the females (Table S1). Taste preference, detection thresholds, and reactivity to taste stimuli may differ between males and females [33]. Fischer et al. [34] reported significant differences in the recognition of bitter, sweet, salty, and sour tastes between men and women in their large epidemiological study; however, other studies did not show any sex-specific differences in taste sensitivity [35]. The exact nature of sex-specific differences in taste sensitivity remains undetermined, and the reported results are not consistent. Moreover, the results of previous studies on the association between taste sensitivity, age, and sex are heterogeneous.

No significant association between taste sensitivity and oral dryness was observed in the present study. This result is consistent with that of the study conducted by Weiffenbach [36]. Matsuo [37] reported at the very least, salivary water and electrolytes are necessary for maintaining taste sensitivity. We measured salivary water on the central area of the dorsum of the tongue. Notably, it is possible that both the amount and composition of saliva affect taste sensitivity.

In this study, the use of oral prosthetic devices, which might affect taste disorders, was not evaluated. However, there are various reports regarding taste and prosthesis use, and there is no uniformity. Har-Zion G. et al. reported that denture wearing did not affect gustatory and olfactory senses [38]. Additionally, Kapur et al. showed that complete artificial dentition may improve taste discrimination and recognition for sweet and sour solutions [39]. Most studies on the relationship between taste and denture usage have assessed upper full dentures. Further research is needed on the effects of denture use on taste.

Pronunciations of /pa/, /ta/, and /ka/ are used to evaluate motor functions of lip movement, the anterior region of the tongue, and the posterior region of the tongue, respectively [9]. In the present study, poor tongue-lip motor function was associated with low taste sensitivity, after adjusting for confounding factors (Table 1). The subgroup analysis conducted using two age categories revealed an association between tongue–lip motor function and taste sensitivity, but only in participants aged <65 years old (Table 2). Kikutani et al. [40] reported that individuals with posterior occlusal support aged 65–88 years showed no significant age-related decline in the pronunciation of the /ta/ or /ka/ syllables.

In the present study, tongue motor function was evaluated based on motor speed and the ODK score of pronouncing /ta/ and /ka/. The results showed that taste sensitivity is independently associated with hypo ODK /ta/ and weakly associated with hypo ODK /ka/. In previous studies, tongue motor function was assessed using oral diadochokinesis in producing the /ta/ sound [7,11]. These findings are consistent with those reported in previous studies. Previous studies have shown that the fungiform, foliate, and circumvallate papillae, known as the gustatory papillae, contain taste buds and function as sensory organs [41,42]. Considering that fungiform papillae are concentrated near the tip of the tongue [43,44], which is used in the pronunciation of the /ta/ syllable, our findings indicate that taste sensitivity is associated with tongue motor function, particularly that of the anterior tongue.

The sense of taste exhibits sensory adaptation similar to those of other senses [45]. Stimulus material is continuously moved over the tongue surface through mouth movements made during eating. Consequently, previously stimulated and adapted parts of the tongue may recover from adaptation while new areas are stimulated [46,47,48]. It seems reasonable to consider that tongue movements may prevent complete adaptation to tastes. Thus, one of the reasons for low taste sensitivity may be poor tongue motor function during taste testing.

It is important to note that the concepts of oral frailty and hypofunction were first described in Japan, which has a homogenous population in terms of ethnic background and language. Most of the instruments used for measuring oral frailty and hypofunction are exclusively available in Japan and were validated using Japanese sample populations [49,50]. Therefore, the assessment of ODK using tools available in other languages needs to be considered. The training of tongue–lip motor function is more likely to be effective [51,52,53]. Thus, it is important to ensure continuous tongue–lip motor function training to maintain optimal oral function [54].

The rate of oral hypofunction among ≥ 65 years olds living in Japanese communities is 43.6% [55]. Tongue–lip motor function training without using an apparatus may help prevent frailty caused by decreased taste sensitivity and anorexia. However, further studies are required to confirm whether tongue–lip motor function training can improve taste sensitivity.

Because this study was mainly designed to analyze the presence or absence of a correlation, we did not set the primary outcome or calculate the sample size. It is important to calculate the sample size before conducting research to compare the differences in tongue–lip motor function and taste test results between groups.

This study has some limitations. First, variations in the time of the taste tests and environment in which they were conducted may have affected the taste sensitivity results. Second, the presence of systemic diseases, the use of medications related to taste disorders, and social determinants were not investigated [56,57,58,59]. Third, the sample size was not calculated prior to the commencement of the study. Fourth, this study is to be a cross-sectional study; thus, we can only hypothesize an association between taste sensitivity and tongue–lip motor function. These limitations should be taken into account when interpreting the results of this study.

## 5. Conclusions

This study demonstrated that decreased taste sensitivity is associated with tongue–lip motor function. Therefore, the early maintenance of oral function and the early testing of taste sensitivity may be beneficial when deciding whether tongue–lip motor function training is effective in taste sensitivity.

## Figures and Tables

**Table 1:** Multiple logistic regression models for the association between taste sensitivity and oral function based on oral diadochokinesis scores.

Outcome = Taste Sensitivity “Incorrect”									
Variable		1% Sweet (n = 32)		0.3% Salty (n = 40)		0.03% Umami (n = 82)		0.1% Umami (n = 38)	
		OR	95% CI	OR	95% CI	OR	95% CI	OR	95% CI
Hypo tongue–lip motor function		3.15	(1.12–8.84)	1.59	(0.66–3.83)	2.05	(1.00–4.28)	1.91	(0.77–4.73)
ODK	/pa/ (<6 times/s)	4.32	(1.70–10.99)	0.89	(0.38–2.13)	1.57	(0.75–3.28)	2.30	(0.97–5.45)
	/ta/ (<6 times/s)	2.33	(0.91–5.97)	1.55	(0.67–3.58)	1.37	(0.65–2.86)	2.98	(1.22–7.30)
	/ka/ (<6 times/s)	1.46	(0.57–3.78)	1.37	(0.58–3.22)	2.11	(1.02–4.40)	1.96	(0.79–4.84)

Notes: CI = confidence interval; OR = odds ratio; ODK = oral diadochokinesis; models are adjusted for age, sex, body mass index, number of teeth, and smoking.

**Table 2:** Multiple logistic regression models for the association between taste sensitivity and oral function based on the oral diadochokinesis in two age groups (≥65 years <65 years).

Outcome = Taste Sensitivity “Incorrect”									
Variable		≥65 years							
		1% Sweet (n = 20)		0.3% Salty (n = 32)		0.03% Umami (n = 50)		0.1% Umami (n = 25)	
		OR	95% CI	OR	95% CI	OR	95% CI	OR	95% CI
Hypo tongue–lip motor function		1.91	(0.54–6.78)	1.09	(0.41–2.89)	2.17	(0.87–5.46)	1.13	(0.40–3.19)
ODK	/pa/ (<6 times/s)	2.85	(0.93–8.70)	0.60	(0.23–1.59)	1.44	(0.61–3.40)	1.87	(0.71–4.93)
	/ta/ (<6 times/s)	1.77	(0.60–5.26)	1.03	(0.41–2.58)	1.16	(0.50–2.67)	1.92	(0.71–5.15)
	/ka/ (<6 times/s)	1.14	(0.37–3.57)	1.20	(0.47–3.09)	1.88	(0.79–4.46)	1.15	(0.42–3.14)
Variable		<65 years							
		1% Sweet (n = 12)		0.3% Salty (n = 8)		0.03% Umami (n = 32)		0.1% Umami (n = 13)	
		OR	95% CI	OR	95% CI	OR	95% CI	OR	95% CI
Hypo tongue–lip motor function		7.47	(1.35–41.48)	6.14	(1.04–36.17)	2.46	(0.69–8.73)	5.46	(1.01–29.36)
ODK	/pa/ (<6 times/s)	30.85	(3.76–252.83)	4.05	(0.67–24.63)	3.70	(0.60–22.72)	4.03	(0.58–27.84)
	/ta/ (<6 times/s)	6.45	(0.93–44.88)	14.28	(1.77–115.05)	9.84	(0.98–99.00)	11.98	(1.32–108.65)
	/ka/ (<6 times/s)	2.58	(0.56–11.85)	4.11	(0.78–21.55)	2.72	(0.66–11.26)	10.50	(1.60–69.06)

Notes: CI = confidence interval; OR = odds ratio; ODK = oral diadochokinesis; models are adjusted for sex, body mass index, number of teeth, and smoking.

## Data Availability

The data presented in this study are available upon request from the corresponding author.

## Supplementary Materials

The following supporting information can be downloaded at: https://www.mdpi.com/article/10.3390/jcm13164711/s1. Table S1: Characteristics of the study population according to the results of the taste sensitivity test. Table S2: Association between taste sensitivity and oral function status.

## References

[1] Population Projections for Japan (2023 Estimates) Summary of Results. https://www.ipss.go.jp/pp-zenkoku/j/zenkoku2023/pp2023_gaiyou.pdf.

[2] Tomita H., Ikeda M., Okuda Y. (1986). Basis and practice of clinical taste examination. Auris Nasus Larynx.

[3] Nin T., Tanaka M., Nishida K., Yamamoto J., Miwa T. (2022). A clinical survey on patients with taste disorders in Japan: A comparative study. Auris Nasus Larynx.

[4] Nin T., Tsuzuki K. (2024). Diagnosis and treatment of taste disorders in Japan. Auris Nasus Larynx.

[5] Whitelock E., Ensaff H. (2018). On your own: Older adults’ food choice and dietary habits. Nutrients.

[6] Iwasaki M., Hirano H., Ohara Y., Motokawa K. (2021). The association of oral function with dietary intake and nutritional status among older adults: Latest evidence from epidemiological studies. Jpn Dent. Sci. Rev..

[7] Tanaka T., Takahashi K., Hirano H., Kikutani T., Watanabe Y., Ohara Y., Furuya H., Tetsuo T., Akishita M., Iijima K. (2018). Oral frailty as a risk factor for physical frailty and mortality in community-dwelling elderly. J. Gerontol. A Biol. Sci. Med. Sci..

[8] Botelho J., Mascarenhas P., Viana J., Proença L., Orlandi M., Leira Y., Chambrone L., Mendes J.J., Machado V. (2022). An umbrella review of the evidence linking oral health and systemic noncommunicable diseases. Nat. Commun..

[9] Russell C.A. (2007). The impact of malnutrition on healthcare costs and economic considerations for the use of oral nutritional supplements. Clin. Nutr. Suppl..

[10] Kamimura K., Okamoto S., Shiraishi K., Sumita K., Komamura K., Tsukao A., Kuno S. (2023). Financial incentives for exercise and medical care costs. Int. J. Econ. Policy Stud..

[11] Minakuchi S., Tsuga K., Ikebe K., Ueda T., Tamura F., Nagao K., Furuya J., Matsuo K., Yamamoto K., Kanazawa M. (2018). Oral hypofunction in the older population: Position paper of the Japanese Society of Gerodontology in 2016. Gerodontology.

[12] Tanaka T., Hirano H., Ikebe K., Ueda T., Iwasaki M., Shirobe M., Minakuchi S., Akishita M., Arai H., Iijima K. (2023). Oral frailty five-item checklist to predict adverse health outcomes in community-dwelling older adults: A Kashiwa cohort study. Geriatr. Gerontol. Int..

[13] Iwasaki M., Yoshihara A., Sato M., Minagawa K., Shimada M., Nishimuta M., Ansai T., Yoshitake Y., Miyazaki H. (2018). Dentition status and frailty in community-dwelling older adults: A 5-year prospective cohort study. Geriatr. Gerontol. Int..

[14] Kida S., Aoyama N., Fujii T., Taniguchi K., Yata T., Iwane T., Yamamoto T., Tamaki K., Minabe M., Komaki M. (2023). Influence of meal sequence and number of teeth on nutrient intake: A cross-sectional study. Nutrients.

[15] Fujii T., Aoyama N., Kida S., Taniguchi K., Yata T., Minabe M., Komaki M. (2023). Associations between Periodontal Status and Liver Function in the Japanese Population: A Cross-Sectional Study. J. Clin. Med..

[16] Taniguchi K., Aoyama N., Fujii T., Kida S., Yata T., Takeda A.K., Minabe M., Komaki M. (2024). Oral and Intestinal Bacterial Flora in Patients with Increased Periodontal Inflamed Surface Area: A Cross-Sectional Study. J. Clin. Med..

[17] Ohta M., Imamura Y., Chebib N., Schulte-Eickhoff R.M., Allain S., Genton L., Mojon P., Graf C., Ueda T., Muller F. (2022). Oral function and nutritional status in non-acute hospitalised elders. Gerodontology.

[18] Wang J.J., Liang K.L., Lin W.J., Chen C.Y., Jiang R.S. (2020). Influence of age and sex on taste function of healthy subjects. PLoS ONE.

[19] Gudziol H., Hummel T. (2007). Normative values for the assessment of gustatory function using liquid tastants. Acta Otolaryngol..

[20] Cecchini M.P., Fasano A., Boschi F., Osculati F., Tinazzi M. (2015). Taste in Parkinson’s disease. J. Neurol..

[21] Welge-Lussen A., Dörig P., Wolfensberger M., Krone F., Hummel T. (2011). A study about the frequency of taste disorders. J. Neurol..

[22] Nishibori S. (2013). Masuta Chorigaku: Food and Health.

[23] Furukawa H. (1991). Synergistic effects in the taste of α-amino-dicarboxylates and L-homocysteinate with 5’-Inosinat. BBB.

[24] Zocchi D., Wennemuth G., Oka Y. (2017). The cellular mechanism for water detection in the mammalian taste system. Nat. Neurosci..

[25] Mizuta E. (2015). Impact of taste sensitivity on lifestyle-related diseases. Yakugaku Zasshi.

[26] Kanda Y. (2001). Salt taste acuity and related factors in the community elderly. J. Kyorin. Med. Soc..

[27] Saito S., Takama F., Toyomaki T. (1972). The effect of the different environment on the taste. Jpn J. Nutr Diet..

[28] Schiffman S.S., Gatlin C.A. (1993). Clinical physiology of taste and smell. Annu. Rev. Nutr..

[29] Weiffenbach J.M., Cowart B.J., Baum B.J. (1986). Taste intensity perception in aging. J. Gerontol..

[30] Methven L., Allen V.J., Withers C.A., Gosney M.A. (2012). Ageing and taste. Proc. Nutr. Soc..

[31] Barragán R., Coltell O., Portolés O., Asensio E.M., Sorlí J.V., Ortega-Azorín C., Gonzalez J.I., Saiz C., Fernandez-Carrion R., Ordovas J.M. (2018). Bitter, sweet, salty, sour and umami taste perception decreases with age: Sex-specific analysis, modulation by genetic variants and taste-preference associations in 18 to 80 year-old subjects. Nutrients.

[32] Doyle M.E., Premathilake H.U., Yao Q., Mazucanti C.H., Egan J.M. (2023). Physiology of the tongue with emphasis on taste transduction. Physiol Rev..

[33] Martin L.J., Sollars S.I. (2017). Contributory role of sex differences in the variations of gustatory function. J. Neurosci. Res..

[34] Fischer M.E., Cruickshanks K.J., Schubert C.R., Pinto A., Klein B.E.K., Klein R., Nieto F.J., Pankow J.S., Huang G.-H., Snyder D.J. (2013). Taste intensity in the Beaver Dam Offspring Study. Laryngoscope.

[35] Mennella J.A., Bobowski N.K. (2015). The sweetness and bitterness of childhood: Insights from basic research on taste preferences. Physiol. Behav..

[36] Weiffenbach J.M., Schwartz L.K., Atkinson J.C., Fox P.C. (1995). Taste performance in Sjogren’s syndrome. Physiol. Behav..

[37] Matsuo R. (2000). Role of saliva in the maintenance of taste sensitivity. Crit. Rev. Oral Biol. Med..

[38] Har-Zion G., Brin I., Steiner J. (2004). Psychophysical testing of taste and flavour reactivity in young patients undergoing treatment with removable orthodontic appliances. Eur. J. Orthod..

[39] Kapur K.K., Collister T., Fisher E.E. (1967). Masticatory and gustatory salivary reflex secretion rates and taste thresholds of denture wearers. J. Prosthet. Dent..

[40] Kikutani T., Tamura F., Nishiwaki K., Kodama M., Suda M., Fukui T., Takahashi N., Yoshida M., Akagawa Y., Kimura M. (2009). Oral motor function and masticatory performance in the community-dwelling elderly. Odontology.

[41] Mbiene J.P., Mistretta C.M. (1997). Initial innervation of embryonic rat tongue and developing taste papillae: Nerves follow distinctive and spatially restricted pathways. Acta. Anat..

[42] Mbiene J.P., Maccallum D.K., Mistretta C.M. (1997). Organ cultures of embryonic rat tongue support tongue and gustatory papilla morphogenesis in vitro without intact sensory ganglia. J. Comp. Neurol..

[43] Jung H.S., Akita K., Kim J.K. (2004). Spacing patterns on tongue surface-gustatory papilla. Int. J. Dev. Biol..

[44] Kobayashi K., Kumakura M., Yoshimura K., Takahashi M., Zeng J.H., Kageyama I., Kobayashi K. (2004). Comparative morphological studies on the stereo structure of the lingual papillae of selected primates using scanning electron microscopy. Ann. Anat..

[45] McBurney D.H. (2011). Taste and olfaction: Sensory discrimination. Compr Physiol..

[46] Gent J.F., McBurney D.H. (1978). Time course of gustatory adaptation. Percept. Psychophys..

[47] O’Mahony M., Wong S.Y. (1989). Time-intensity scaling with judges trained to use a calibrated scale: Adaptation, salty and umami tastes. J. Sens. Stud..

[48] Theunissen M.J., Kroeze J.H., Schifferstein H.N. (2000). Method of stimulation, mouth movements, concentration, and viscosity: Effects on the degree of taste adaptation. Percept. Psychophys..

[49] Schimmel M., Domioni T., Bukvic H., Arakawa I., Seifert E., Abou-Ayash S. (2022). Oral diadochokinesis and associated oro-facial function in young and old German mother-tongue speakers: A cross-sectional study. Gerodontology.

[50] Ben-David B.M., Icht M. (2017). Oral-diadochokinetic rates for Hebrew-speaking healthy ageing population: Non-word versus real-word repetition. Int. J. Lang. Commun. Disord..

[51] Shirobe M., Watanabe Y., Tanaka T., Hirano H., Kikutani T., Nakajo K., Sato T., Furuya J., Minakuchi S., Iijima K. (2022). Effect of an oral frailty measures program on community-dwelling elderly people: A cluster-randomized controlled trial. Gerontology.

[52] Sakayori T., Maki Y., Hirata S., Okada M., Ishii T. (2013). Evaluation of a Japanese “Prevention of Long-term Care” project for the improvement in oral function in the high-risk elderly. Geriatr. Gerontol. Int..

[53] Hatanaka Y., Furuya J., Sato Y., Uchida Y., Osawa T., Shichita T., Suzuki H., Minakuchi S. (2022). Impact of oral health guidance on the tongue-lip motor function of outpatients at a dental hospital. Gerodontology.

[54] Sakayori T., Maki Y., Ohkubo M., Ishida R., Hirata S., Ishii T. (2016). Longitudinal evaluation of community support project to improve oral function in Japanese elderly. Bull. Tokyo Dent. Coll..

[55] Kugimiya Y., Watanabe Y., Ueda T., Motokawa K., Shirobe M., Igarashi K., Hoshino D., Takano T., Sakurai K., Taniguchi Y. (2020). Rate of oral frailty and oral hypofunction in rural community-dwelling older Japanese individuals. Gerodontology.

[56] Risso D., Drayna D., Morini G. (2020). Alteration, reduction and taste loss: Main causes and potential implications on dietary habits. Nutrients.

[57] Menni C., Valdes A.M., Freidin M.B., Sudre C.H., Nguyen L.H., Drew D.A., Ganesh S., Varsavsky T., Cardoso M.J., El-Sayed Moustafa J.S. (2020). Real-time tracking of self-reported symptoms to predict potential COVID-19. Nat. Med..

[58] Tsuchiya H. (2023). Treatments of COVID-19-associated taste and saliva secretory disorders. Dent. J..

[59] Błochowiak K. (2022). Smell and Taste Function and Their Disturbances in Sjögren’s Syndrome. Int. J. Environ. Res. Public Health.

